# Association of Serum Bilirubin Levels With Histopathological Severity of Diabetic Nephropathy in Patients With Type 2 Diabetes: A Cross‐Sectional Biopsy Study

**DOI:** 10.1155/jdr/2435997

**Published:** 2026-07-23

**Authors:** Xiaorong Wang, Congqin Zhang, Chunxia Zhang, Yongmei Hao, Lingling Xing, Chang Guo, Lin Mu

**Affiliations:** ^1^ Department of Nephrology, The Second Hospital of Hebei Medical University, Shijiazhuang, Hebei, China, hebmu.edu.cn; ^2^ Department of Endocrinology, The Second Hospital of Hebei Medical University, Shijiazhuang, Hebei, China, hebmu.edu.cn; ^3^ Department of Medical Oncology, Affiliated Hospital of Hebei University, Baoding, Hebei, China, hbu.cn

**Keywords:** diabetic nephropathy, histopathological severity, renal biopsy, total bilirubin, Type 2 diabetes mellitus

## Abstract

**Background:**

Prior studies have suggested that serum total bilirubin (TBil) may be linked to diabetic nephropathy (DN); however, its potential relationship with structural renal injury in histopathology has not been well defined.

**Objective:**

The objective of the study is to explore whether physiological levels of TBil are associated with the severity of renal pathological changes in individuals with Type 2 diabetes mellitus (T2DM) and biopsy‐confirmed DN.

**Methods:**

A cross‐sectional cohort of 401 individuals with T2DM and biopsy‐confirmed DN was included. DN severity was determined according to the Renal Pathology Society classification and categorized as early‐stage DN (Classes I–II) or advanced DN (Classes III–IV). Multivariable logistic regression was used to evaluate the association between TBil and advanced DN. TBil quartiles, restricted cubic spline modeling, subgroup and sensitivity analyses, and receiver operating characteristic curve analysis were also performed.

**Results:**

Among 401 biopsy‐confirmed DN patients, 258 (64.3%) were classified as having advanced DN. In multivariable models, higher TBil levels were independently associated with lower odds of advanced DN after full adjustment (OR = 0.882, 95% CI 0.827–0.941; *p* < 0.001). When TBil was analyzed by quartiles, individuals in the highest quartile exhibited significantly lower odds of advanced‐stage disease relative to those in the lowest quartile in the fully adjusted model (OR = 0.331, 95% CI 0.153–0.718; *p* = 0.005), with a notable dose–response pattern across quartiles (*p* for trend = 0.003). Restricted cubic spline analyses confirmed an inverse association between TBil levels and advanced DN. Receiver operating characteristic curve analysis identified a TBil cutoff of 9.9 *μ*mol/L for discriminating advanced DN, with an area under the curve of 0.684. The results were broadly consistent across subgroup and sensitivity analyses.

**Conclusion:**

Higher physiological TBil levels were linked to lower histopathological severity of DN in patients with Type 2 diabetes.

## 1. Introduction

Type 2 diabetes mellitus (T2DM) has emerged as a major global public health challenge with a rising incidence, and diabetic nephropathy (DN) stands out as one of its most devastating chronic microvascular complications [1]. With the expanding population affected by diabetes, DN now accounts for a substantial proportion of kidney failure and contributes markedly to the worldwide burden of chronic kidney disease [2, 3]. Although routine clinical indicators—including estimated glomerular filtration rate (eGFR) and urinary albumin (ALB) excretion—are commonly used to assess disease severity, renal histopathological assessment remains essential for characterizing the underlying pathological burden and disease progression in DN.

The development of DN involves multiple interacting processes, including metabolic disturbance, hemodynamic stress, inflammation, genetic susceptibility, and oxidative injury [4]. Among these mechanisms, oxidative stress induced by chronic hyperglycemia may promote both glomerular and tubulointerstitial damage, contributing to pathological changes such as mesangial expansion, glomerulosclerosis, and interstitial fibrosis [5]. Identifying endogenous factors that may modulate oxidative stress–related renal damage could therefore provide insights into the heterogeneity of histopathological severity observed among patients with DN.

Bilirubin, traditionally regarded as a byproduct of heme metabolism, has been increasingly recognized as an endogenous antioxidant since the seminal work by Stocker et al. in 1987 [6]. Both experimental and clinical studies have linked mildly higher bilirubin concentrations to lower oxidative burden and reduced risk of several cardiometabolic disorders [7, 8]. In the context of DN, accumulating clinical evidence suggests that lower physiological TBil levels may be related to the occurrence and progression of DN [9, 10]. These observations are further supported by experimental studies demonstrating that bilirubin administration can attenuate renal injury and improve renal histopathological changes in diabetic animal models [11].

Despite the accumulating evidence linking TBil levels to DN, data on its association with renal histopathological severity remain limited. In particular, few studies have examined this relationship in biopsy‐proven DN cohorts or explored its correlations with specific pathological features. Therefore, this retrospective cross‐sectional study was designed to examine whether physiological TBil levels differ according to renal histopathological severity in patients with biopsy‐confirmed DN.

## 2. Materials and Methods

### 2.1. Study Design and Participants

Patients with T2DM who underwent native kidney biopsy were retrospectively identified from the Second Hospital of Hebei Medical University. This cross‐sectional analysis covered the period from January 2015 to August 2025.

The institutional review board of the Second Hospital of Hebei Medical University (Approval No. 2025‐R642) granted permission for the conduct of this retrospective study, which conforms to the principles of the Declaration of Helsinki. The data were obtained from inpatient electronic medical records. As only deidentified clinical data were used in this retrospective analysis, the requirement for written informed consent was waived.

**Figure 1:**
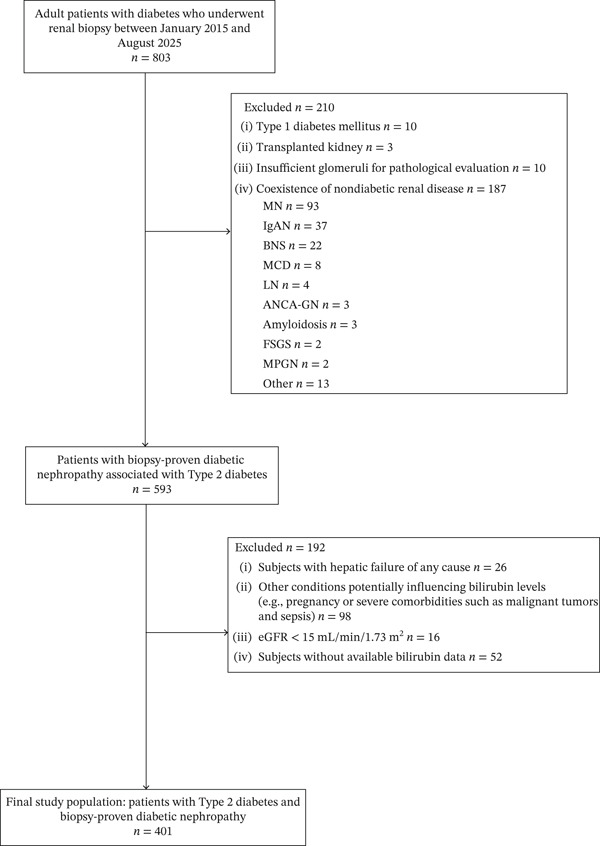
Flow diagram of patient screening and inclusion. Abbreviations: MN, membranous nephropathy; IgAN, IgA nephropathy; BNS, benign nephrosclerosis; MCD, minimal change disease; LN, lupus nephritis; ANCA‐GN, antineutrophil cytoplasmic antibody–associated glomerulonephritis; FSGS, focal segmental glomerulosclerosis; MPGN, membranoproliferative glomerulonephritis; eGFR, estimated glomerular filtration rate.

Kidney biopsy was performed based on institutional practice and clinical indications, with reference to current recommendations for renal biopsy among diabetic patients [12]. Individuals were not eligible for inclusion if any of the following conditions were present: (1) Type 1 diabetes or other diabetes subtypes; (2) history of kidney transplantation, insufficient glomerular count for reliable pathological assessment, or concurrent nondiabetic kidney disease; (3) eGFR < 15 mL/min/1.73 m^2^; (4) hepatic failure of any etiology or other conditions that could affect bilirubin concentrations, including pregnancy, malignant tumors, or sepsis; or (5) unavailable bilirubin data. After screening according to predefined eligibility criteria, 401 patients with biopsy‐confirmed DN were retained for the final analysis. The participant selection process is summarized in Figure 1.

### 2.2. Clinical and Laboratory Evaluation

Demographic and clinical information, including sex, age, body mass index (BMI), systolic blood pressure (SBP), smoking status, duration of diabetes, history of diabetic retinopathy (DR, yes or no), and medication use (ACEI/ARB and SGLT2i, yes or no), was obtained during hospitalization. Fasting venous blood samples were collected from all participants for biochemical and hematological analyses. Serum biochemical parameters, including total bilirubin (TBil; reference range: 0–26 *μ*mol/L), ALB, glycosylated hemoglobin (HbA1c), creatinine (Scr), uric acid (UA), total cholesterol (TC), triglyceride (TG), low‐density lipoprotein cholesterol (LDL‐C), and high‐density lipoprotein cholesterol (HDL‐C), were measured using standard automated laboratory methods. Neutrophil‐to‐lymphocyte ratio (NLR) was calculated as neutrophil count divided by lymphocyte count.

Urinary protein excretion over 24 h was recorded (grams/24 h). Renal function was assessed by eGFR, calculated using the 2009 CKD‐EPI creatinine equation [13].

### 2.3. Renal Pathological Examination

Standard pathological evaluation included light microscopy and immunofluorescence, which were routinely applied to all samples. Electron microscopy (EM) was applied for cases in which light microscopy raised suspicion of early DN or when further evaluation was required to clarify the diagnosis.

DN was staged from Class I to Class IV using the Renal Pathology Society (RPS) criteria [14]. In the main analysis, Classes I–II were grouped as early‐stage disease, while Classes III–IV were considered advanced‐stage DN. Tubulointerstitial and vascular changes were graded semiquantitatively, including interstitial fibrosis and tubular atrophy (IFTA; 0–3), interstitial inflammation (0–2), arteriolar hyalinosis (0–2), and arteriosclerosis (0–2), following the standard pathological scoring protocol [14].

### 2.4. Outcomes

Advanced DN was defined as RPS Classes III–IV and served as the primary outcome.

### 2.5. Statistical Analysis

The data were processed using R software, Version 4.5.1, and SPSS software, Version 27.0. The distribution of continuous variables was evaluated using the Shapiro–Wilk test. Continuous variables with a normal distribution are summarized as mean ± standard deviation, whereas nonnormally distributed variables are presented as median with interquartile range (IQR). Continuous variables were analyzed using either independent‐samples *t*‐tests or Mann–Whitney *U* tests according to distributional characteristics. For categorical data, chi‐square tests or Fisher′s exact tests were applied as appropriate.

The association between serum TBil and advanced DN (Classes III–IV) was assessed using logistic regression models, with results expressed as odds ratios (ORs) and 95% confidence intervals (CIs). Covariates were adjusted in three sequential models: Model 1 (age, sex, BMI, smoking status, and duration of diabetes); Model 2 additionally incorporated clinical factors (SBP, HbA1c, ACEI/ARB, and SGLT2i use). Model 3 further adjusted for biochemical and renal parameters (UA, ALB, NLR, lipid profiles, and ln‐transformed 24‐h urinary protein). The proportion of missing data was minimal; therefore, complete‐case analysis was applied in all multivariable regression models.

TBil was additionally analyzed by quartiles, and trend analysis was performed using median values. Restricted cubic spline analysis was used to evaluate potential nonlinear relationships [15]. Stratified and sensitivity analyses were conducted to assess robustness. Spearman correlation analysis was used to assess associations between TBil and histopathological parameters. ROC analysis was performed to evaluate diagnostic performance of TBil for advanced DN. A two‐sided *p* < 0.05 was considered statistically significant.

## 3. Results

### 3.1. Baseline Characteristics of Participants

Basic characteristics according to DN pathological severity are presented in Table 1. Of the 401 eligible patients, 143 were assigned to the early DN group, whereas 258 were assigned to the advanced DN group. Overall, the cohort comprised 266 men (66.3%), and the mean age was 52.95 ± 11.30 years.

**Table 1:** Basic characteristics according to DN pathological severity.

Characteristic	All (*n* = 401)	Early DN (Classes I–II, *n* = 143)	Advanced DN (Classes III–IV, *n* = 258)	SMD	*p*
Age (years)	52.95 ± 11.30	53.14 ± 11.65	52.85 ± 11.04	0.003	0.974
Male, *n* (%)	266 (66.3)	98 (68.5)	168 (65.1)	0.073	0.488
BMI (kg/m^2^)	24.21 (22.84, 25.69)	24.44 (22.76, 25.78)	24.03 (22.84, 25.41)	0.101	0.373
Duration of diabetes (years)	7.00 (2.00, 12.00)	5.00 (1.00, 10.00)	8.00 (3.00, 13.00)	0.283	0.015
SBP (mmHg)	149.92 ± 23.54	146.42 ± 23.45	151.87 ± 23.41	0.212	0.043
Cigarette smoking, *n* (%)	151 (37.7)	47 (32.9)	104 (40.3)	0.155	0.141
HbA1c (%)	7.30 (6.40, 8.75)	7.20 (6.40, 8.70)	7.30 (6.30, 8.90)	0.095	0.761
DR, *n* (%)	245 (62.5)	58 (40.8)	187 (74.8)	0.732	< 0.001
ACEI/ARB, *n* (%)	194 (48.5)	74 (51.7)	120 (46.7)	0.101	0.332
SGLT2i, *n* (%)	166 (41.5)	64 (45.1)	102 (39.5)	0.112	0.282
eGFR (mL·min^−1^·1.73 m^−2^)	59.06 (36.63, 84.81)	75.61 (50.59, 97.97)	49.76 (32.91, 73.23)	0.722	< 0.001
UA (*μ*mol/L)	337.00 (278.00, 386.60)	334.50 (275.00, 394.50)	338.00 (281.00, 384.00)	0.032	0.991
ALB (g/L)	32.00 (27.00, 37.45)	36.90 (32.20, 40.50)	29.95 (25.85, 34.43)	0.925	< 0.001
TC (mmol/L)	4.59 (3.87, 5.64)	4.32 (3.70, 5.23)	4.77 (3.93, 5.84)	0.392	0.001
TG (mmol/L)	1.65 (1.24, 2.35)	1.81 (1.25, 2.60)	1.60 (1.23, 2.23)	0.055	0.062
HDL‐C (mmol/L)	1.00 (0.85, 1.26)	1.00 (0.83, 1.17)	1.01 (0.86, 1.33)	0.220	0.117
LDL‐C (mmol/L)	2.99 (2.35, 3.88)	2.72 (2.21, 3.46)	3.18 (2.45, 4.03)	0.374	0.001
NLR	2.30 (1.76, 3.12)	2.19 (1.63, 2.87)	2.40 (1.85, 3.30)	0.116	0.024
24‐h UP (g/24 h)	3.40 (1.14, 6.74)	1.18 (0.46, 3.66)	4.39 (2.36, 9.76)	0.918	< 0.001
TBil (*μ*mol/L)	6.80 (4.30, 10.40)	9.50 (5.90, 12.30)	6.00 (3.90, 8.40)	0.681	< 0.001

*Note:* Continuous variables were compared using an independent‐samples *t*‐test and categorical variables using chi‐square test or Fisher′s exact test.

Abbreviations: 24‐h UP, 24‐h urinary protein; ACEI/ARB, angiotensin‐converting enzyme inhibitors/angiotensin II receptor blockers; ALB, albumin; BMI, body mass index; DN, diabetic nephropathy; DR, diabetic retinopathy; eGFR, estimated glomerular filtration rate; HDL‐C, high‐density lipoprotein cholesterol; LDL‐C, low‐density lipoprotein cholesterol; NLR, neutrophil‐to‐lymphocyte ratio; SBP, systolic blood pressure; SGLT2i, sodium‐glucose cotransporter‐2 inhibitors; TBil, total bilirubin; TC, total cholesterol; TG, triglyceride; UA, uric acid.

Patients with advanced DN exhibited a longer history of diabetes (8.00 [3.00–13.00] vs. 5.00 [1.00–10.00] years, *p* = 0.015) and a markedly higher frequency of DR (74.8% vs. 40.8%, *p* < 0.001) than those with early DN. In addition, this group showed higher SBP, lower eGFR, reduced serum ALB levels, increased NLR, and greater 24‐h urinary protein excretion. Notably, compared with the early DN group, patients with advanced DN had significantly lower serum TBil levels (6.00 [3.90–8.40] vs. 9.50 [5.90–12.30] *μ*mol/L, *p* < 0.001).

### 3.2. Association of TBil With Advanced DN

The relationship between serum TBil and advanced DN was evaluated using logistic regression models (Table 2). In the initial adjustment model, which included age, sex, BMI, smoking status, and diabetes duration, TBil showed a significant inverse association with disease severity. Each 1 *μ*mol/L increase in TBil was associated with lower odds of advanced DN (OR = 0.845, 95% CI 0.799–0.894; *p* < 0.001).

**Table 2:** Multivariable logistic regression analyses of the association between TBil and advanced DN.

Model	OR (95% CI)	*p*
Model 1	0.845 (0.799–0.894)	< 0.001
Model 2	0.843 (0.795–0.893)	< 0.001
Model 3	0.882 (0.827–0.941)	< 0.001

*Note:* Model 1 included age, sex, BMI, smoking status, and duration of diabetes. Model 2 additionally accounted for SBP and HbA1c, as well as the use of ACEI/ARB and SGLT2i. Model 3 was defined as the fully adjusted model, further incorporating serum UA, ALB, NLR, TC, TG, HDL‐C, LDL‐C, and ln‐UP. Advanced DN was defined as Renal Pathology Society Classes III–IV.

This association remained stable after further adjustment for SBP, HbA1c, and medication use (ACEI/ARB and SGLT2i) in Model 2 (OR = 0.843, 95% CI 0.795–0.893; *p* < 0.001).

When metabolic and renal injury–related variables were additionally incorporated into the fully adjusted model, the association was slightly attenuated but remained statistically significant (OR = 0.882, 95% CI 0.827–0.941; *p* < 0.001).

### 3.3. Dose–Response Association of TBil With Advanced DN Based on Quartiles and RCS Analyses

**Figure 2:**
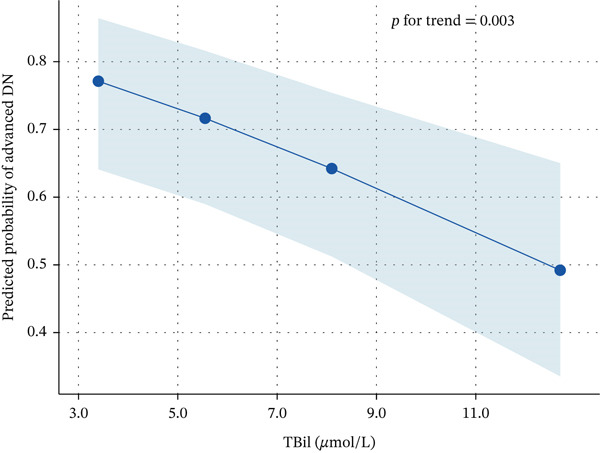
Linear trends in the predicted probability of advanced DN across quartiles of TBil. Points represent estimated probabilities, while shaded regions show the corresponding 95% confidence intervals. The *p* value for trend is shown. Estimates were derived from the fully adjusted multivariable model (Model 3).

**Figure 3:**
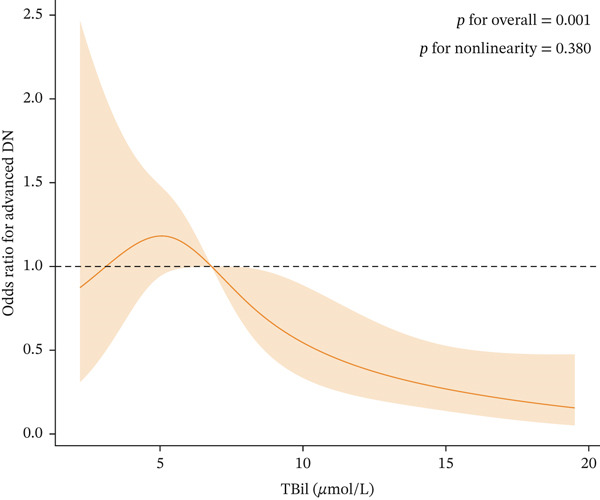
Restricted cubic spline analysis of the association between TBil and advanced DN in the fully adjusted Model 3. The dashed horizontal line indicates an odds ratio of 1.0.

To further explore the association between TBil and advanced DN, TBil levels were categorized into quartiles: Q1 (≤ 4.20), Q2 (4.30–6.76), Q3 (6.80–10.37), and Q4 (≥ 10.40). Baseline clinical and laboratory characteristics according to TBil quartiles are presented in Table S1. The distribution of renal pathological characteristics across TBil quartiles is summarized in Table S2, showing a lower proportion of advanced DN and severe tubulointerstitial lesions at higher TBil levels. Using the lowest quartile as reference, a graded reduction in the odds of advanced DN was observed across increasing TBil categories (Table 3), and this trend persisted after multivariable adjustment (*p* for trend = 0.003) (Figure 2). RCS analysis based on the fully adjusted model further demonstrated a continuous inverse association between TBil and advanced DN (overall *p* = 0.001), without evidence of significant nonlinearity (*p* for nonlinearity = 0.380) (Figure 3).

**Table 3:** Association between TBil quartiles and advanced DN.

	Model 1		Model 2		Model 3	
TBil quartiles (*μ*mol/L)	OR (95% CI)	*p*	OR (95% CI)	*p*	OR (95% CI)	*p*
Q1 (≤ 4.20)	Reference		Reference		Reference	
Q2 (4.30–6.76)	0.785 (0.392–1.570)	0.493	0.806 (0.400–1.627)	0.548	1.353 (0.617–2.967)	0.451
Q3 (6.80–10.37)	0.410 (0.212–0.794)	0.008	0.401 (0.204–0.788)	0.008	0.610 (0.286–1.299)	0.200
Q4 (≥ 10.40)	0.155 (0.080–0.300)	< 0.001	0.155 (0.078–0.307)	< 0.001	0.331 (0.153–0.718)	0.005

*Note:* Model 1 included age, sex, BMI, smoking status, and duration of diabetes. Model 2 additionally accounted for SBP and HbA1c, as well as the use of ACEI/ARB and SGLT2i. Model 3 was defined as the fully adjusted model and additionally included serum UA, ALB, NLR, TC, TG, LDL‐C, HDL‐C, and ln‐UP.

### 3.4. Stratified and Interaction Analyses

Subgroup analyses suggested that the inverse association between TBil and advanced DN was generally consistent across different clinical strata, including renal function, glycemic status, proteinuria level, and diabetes duration (Table S3). No statistically significant interactions were identified (all *p* for interaction > 0.05).

### 3.5. Association of TBil With Interstitial Fibrosis and Inflammation

To further explore the potential link between serum TBil and renal histopathological injury, we evaluated the association of TBil with key tubulointerstitial histopathological features. Spearman correlation analysis indicated that TBil was inversely correlated with both IFTA score (*ρ* = −0.110, *p* = 0.027) and interstitial inflammation score (*ρ* = −0.149, *p* = 0.003).

### 3.6. Sensitivity Analysis

After excluding patients using diuretics at the time of TBil measurement, the fully adjusted model replicated the primary analysis (OR = 0.890, 95% CI 0.834–0.950; *p* < 0.001; Table S4).

As an additional sensitivity analysis, DN severity was analyzed as an ordinal outcome (Classes I–IV) using ordinal logistic regression. This analysis again showed a significant negative association between TBil and DN pathological severity, supporting the consistency of the primary results. However, the Brant test indicated violation of the proportional odds assumption for TBil; therefore, the ordinal logistic regression results were considered exploratory and were used mainly to assess robustness (Table S5).

**Figure 4:**
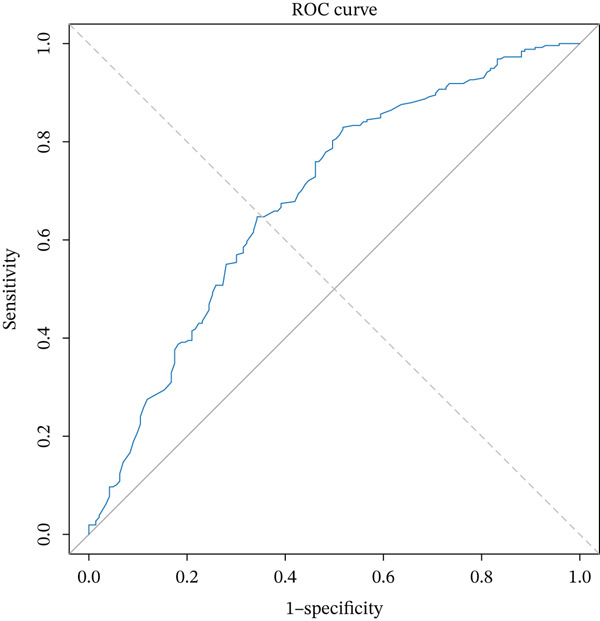
ROC analysis of TBil for discriminating advanced DN in T2DM patients. AUC = 0.684; 95% CI 0.628–0.740; TBIL cutoff = 9.9 * μ*mol/L; Youden index = 0.175; sensitivity = 83.0*%*; specificity = 48.0*%*.

ROC analysis showed that serum TBil had a moderate discriminatory ability for advanced DN (AUC = 0.684; 95% CI 0.628–0.740), with an optimal cutoff value of 9.9 *μ*mol/L (sensitivity 0.83 and specificity 0.48) (Figure 4).

## 4. Discussion

In the present biopsy‐based study of patients with T2DM, lower serum TBil was closely linked to more severe renal pathological involvement in DN. This association remained evident after adjustment for demographic characteristics, metabolic factors, inflammatory markers, lipid profiles, and proteinuria. Moreover, analyses based on TBil quartiles and RCS modeling suggested a graded, dose‐dependent relationship between TBil and renal pathological severity, with progressively lower odds of advanced DN observed at higher physiological TBil concentrations.

Although proteinuria and renal function are widely used to assess the severity and progression of DN, they do not fully capture the underlying histopathological heterogeneity of the disease. In clinical practice, patients with comparable levels of proteinuria may exhibit markedly different degrees of structural renal damage on biopsy. Notably, a subset of patients already demonstrates advanced glomerular and tubulointerstitial lesions despite the absence of extreme proteinuria [16]. These observations underscore the limitations of relying solely on proteinuria or eGFR to reflect underlying intrinsic renal pathology. In this context, our findings suggest that TBil may provide additional information beyond conventional clinical indicators. In the present study, the association between TBil and histopathological severity remained evident after adjustment for traditional risk factors, including proteinuria. This indicates that TBil may capture pathological information not fully reflected by conventional markers, thereby providing complementary insight into disease severity in patients with DN. To date, studies examining the relationship between TBil and renal histopathology in DN remain limited. The present study constitutes one of the largest biopsy‐confirmed cohorts available for evaluating this association.

The association between lower TBil and more severe renal pathological involvement observed in our study is broadly consistent with previous epidemiological evidence. A recent longitudinal study demonstrated that both baseline TBil levels and their temporal decline were independently associated with accelerated renal function deterioration in patients with diabetes [17]. Similarly, a decade‐long observational cohort study conducted in Japan reported that higher baseline TBil concentrations were related to a slower rate of renal function decline over time [18]. Together, these findings are consistent with the observed association between TBil and histopathological severity in the present study and provide supportive epidemiological context for a potential link between TBil levels and structural renal injury in DN.

However, evidence regarding the association between TBil and DN has not been entirely consistent across studies. For instance, analyses based on NHANES data from the United States did not identify a significant association between TBil levels and DN in the overall population with diabetes, despite its large sample size and long follow‐up period [10]. Such discrepancies may reflect substantial heterogeneity related to population characteristics, study design, and outcome definitions. Population‐specific genetic variations in pathways regulating bilirubin metabolism and vascular or inflammatory responses, such as UGT1A1 and NOS3, together with differences in lifestyle and comorbidity profiles, may contribute to the inconsistent findings across studies [19, 20]. These considerations underscore the importance of interpreting associations between TBil and DN within specific clinical and pathological contexts. Recent studies in broader CKD populations have also suggested a potential prognostic role of serum bilirubin in kidney disease progression [21], although external validation in specific CKD subtypes remains limited.

Notably, no significant relationship was detected between TBil and vascular lesions, including arteriolar hyalinosis or arteriosclerosis. This observation aligns with previous biopsy‐based studies [9, 22], which may reflect the predominantly structural and chronic nature of these vascular changes, as they are largely driven by long‐standing hemodynamic and metabolic factors rather than dynamic oxidative or inflammatory modulation.

The biological plausibility of our findings is supported by growing evidence that bilirubin exerts pleiotropic protective effects beyond its conventional role as a byproduct of heme metabolism. Bilirubin is now recognized as a potent endogenous antioxidant, with reported protective associations across multiple organ systems [13–25]. In the context of DN, oxidative stress is a key driver of tubulointerstitial inflammation and fibrotic remodeling, processes that are closely linked to disease progression [5]. Bilirubin has been reported to attenuate angiotensin II–related renal injury by suppressing NADPH oxidase–derived oxidative stress and downstream profibrotic signaling, including HIF‐1*α*–mediated pathways, which may contribute to a reduction in tubulointerstitial fibrosis [26, 27]. At the glomerular level, a preclinical study further suggests that bilirubin may mitigate podocyte injury by suppressing apoptosis‐related pathways and promoting autophagy‐mediated cellular survival, consistent with a biological framework in which lower physiological TBil levels are associated with more severe histopathological injury in DN [27]. Additionally, prior in vitro evidence has indicated that mildly increased bilirubin levels may exert immunomodulatory and anti‐inflammatory actions, including suppression of ER stress–associated inflammatory signaling and reduced release of proinflammatory cytokines such as IL‐6, which may partially contribute to the attenuated interstitial inflammatory activity observed in individuals with higher physiological TBil levels [28].

Of note, emerging evidence suggests that bilirubin influences systemic metabolic regulation beyond its established antioxidant and anti‐inflammatory actions. In animal models, either mildly elevated bilirubin levels or bilirubin administration has been associated with reduced blood glucose and attenuated weight gain, suggesting a role in systemic energy metabolism [29, 30]. Furthermore, in vitro findings indicate that bilirubin suppresses lipid accumulation in hepatocytes through activation of the PPAR*α* pathway, thereby improving hepatic lipid metabolism [31]. Consistently, bilirubin has also been implicated in metabolic dysfunction‐associated steatotic liver disease (MASLD), which is increasingly recognized as a hepatic manifestation of systemic metabolic dysfunction and shares key pathophysiological features with T2DM, including insulin resistance and lipid dysregulation [31, 32]. Collectively, these observations imply that bilirubin may participate in maintaining metabolic balance and could indirectly modulate the onset and progression of metabolic disorders.

Our findings suggest that TBil may provide complementary information for assessing disease severity in patients with DN. Consistent with the limitations of conventional markers discussed above, ROC curve analysis demonstrated a moderate discriminatory ability of TBil for advanced DN (AUC = 0.684), with an optimal cutoff value of 9.9 *μ*mol/L within the physiological reference range. The relatively higher sensitivity but modest specificity further suggests that TBil may be more suitable as a screening rather than a diagnostic biomarker. Given its routine availability, low cost, and standardized measurement, TBil could serve as an adjunctive biomarker to refine risk stratification and inform clinical decision‐making, such as closer monitoring or consideration of early nephrology referral. Importantly, these findings do not support replacing established clinical markers, but rather suggest a potential role for TBil in capturing aspects of intrinsic renal pathology that are not readily reflected by conventional indices alone.

## 5. Limitations and Conclusion

A number of limitations should be acknowledged. First, due to its cross‐sectional design, temporal and causal relationships cannot be established, and the single‐center design may limit generalizability. Second, kidney biopsy is typically performed in patients with more severe, atypical, or clinically uncertain renal involvement; therefore, the study population may not fully represent all patients with T2DM and DN. Third, the absence of longitudinal follow‐up data limited the assessment of the prognostic value of TBil. Despite these limitations, the availability of detailed histopathological data in a relatively large biopsy‐confirmed DN cohort provides a valuable opportunity to explore clinicopathological associations that are difficult to address in population‐based studies.

In conclusion, lower physiological TBil levels were observed in patients with more severe histopathological DN, particularly advanced RPS classes and more pronounced tubulointerstitial injury. These results indicate that TBil may offer additional information for evaluating pathological severity in DN. Further multicenter studies with longitudinal follow‐up are warranted to determine whether TBil has prognostic significance and to elucidate its clinical relevance.

## Author Contributions

Xiaorong Wang: conceptualization, investigation, validation, data curation, and writing—original draft. Congqin Zhang: investigation, data curation, and writing—review and editing. Chunxia Zhang: formal analysis, methodology, software, and writing—review and editing. Yongmei Hao: formal analysis, methodology, software, and writing—review and editing. Lingling Xing: conceptualization, methodology, supervision, and writing—review and editing. Chang Guo: visualization and writing—review and editing. Lin Mu: conceptualization, methodology, supervision, project administration, and writing—review and editing.

## Funding

The study was funded by Hebei Provincial Medical Science Research Project (No. 20220984).

## Disclosure

All authors have read and approved the final version of the manuscript. Lin Mu served as the manuscript guarantor, had full access to all study data, and takes responsibility for the integrity of the data and the accuracy of the data analysis.

## Conflicts of Interest

The authors declare no conflicts of interest.

## Supporting information


**Supporting Information** Additional supporting information can be found online in the Supporting Information section. Table S1 Baseline clinical and laboratory characteristics according to quartiles of TBil. Table S2. Renal pathological characteristics according to TBil quartiles. Table S3. Stratified analyses of the association between TBil and advanced DN. Table S4. Sensitivity analysis of the association between TBil and advanced DN after excluding patients with documented diuretic use. Table S5. Sensitivity analysis of the association between TBil and DN pathological severity using ordinal logistic regression.

## Data Availability

The data supporting the findings of this study are available from the corresponding author upon reasonable request. All data were anonymized to ensure participant confidentiality in accordance with institutional ethical standards.
