# Supplementary material for: Association of Serum Bilirubin Levels With Histopathological Severity of Diabetic Nephropathy in Patients With Type 2 Diabetes: A Cross‐Sectional Biopsy Study
Source: J Diabetes Res. 2026 Jul 23;2026:2435997. doi: 10.1155/jdr/2435997 (PMC13396876; doi:10.1155/jdr/2435997)
Supplement: Supplementary file 1 — Supporting Information Additional supporting information can be found online in the Supporting Information section. Table S1 Baseline clinical and laboratory characteristics according to quartiles of TBil. Table S2. Renal pathological characteristics according to TBil quartiles. Table S3. Stratified analyses of the association between TBil and advanced DN. Table S4. Sensitivity analysis of the association between TBil and advanced DN after excluding patients with documented diuretic use. Table S5. Sensitivity analysis of the association between TBil and DN pathological severity using ordinal logistic regression. [file JDR-2026-2435997-s001.docx]

**Supplementary Table S1.** Baseline clinical and laboratory characteristics according to quartiles of TBil

| **Variable** | **Q1**  **(≤ 4.20, n = 98)** | **Q2**  **(4.30–6.76, n = 102)** | **Q3**  **(6.80–10.37, n = 99)** | **Q4**  **(≥ 10.40, n = 102)** | ***P*** |
| --- | --- | --- | --- | --- | --- |
| Age (years) | 51.62 ± 11.11 | 53.51 ± 12.32 | 52.65 ± 11.04 | 53.94 ± 10.70 | 0.485 |
| Male, n (%) | 56 (57.1) | 67 (65.7) | 67 (67.7) | 76 (74.5) | 0.077 |
| BMI (kg/m²) | 23.34 ± 2.04 | 25.16 ± 3.46 | 24.99 ± 2.83 | 24.83 ± 2.85 | < 0.001 |
| Duration of diabetes (years) | 6.50 (1.75, 12.00) | 8.00 (1.90, 14.00) | 8.00 (2.00, 13.00) | 6.50 (2.75, 10.00) | 0.547 |
| SBP (mmHg) | 151.00 (136.75, 164.00) | 155.00 (136.75, 170.25) | 147.00 (129.00, 168.00) | 143.00 (130.00, 158.00) | 0.013 |
| Cigarette smoking, n (%) | 39 (39.8) | 31 (30.4) | 44 (44.4) | 37 (36.3) | 0.211 |
| HbA1c (%) | 7.10 (6.08, 8.50) | 7.10 (6.10, 8.53) | 7.50 (6.80, 9.00) | 7.60 (6.45, 8.95) | 0.046 |
| DR, n (%) | 66 (68.0) | 75 (75.0) | 54 (55.1) | 50 (51.5) | 0.002 |
| ACEI/ARB, n (%) | 42 (42.9) | 57 (55.9) | 43 (43.9) | 52 (51.0) | 0.207 |
| SGLT2i, n (%) | 32 (32.7) | 43 (42.2) | 45 (45.5) | 46 (45.5) | 0.213 |
| eGFR (mL/min/1.73 m²) | 49.06 (23.61, 82.23) | 50.33 (33.32, 80.76) | 58.03 (39.31, 82.38) | 70.62 (50.03, 97.05) | < 0.001 |
| UA (µmol/L) | 323.50 (270.50, 386.25) | 338.50 (283.50, 396.00) | 340.00 (287.00, 375.00) | 336.00 (275.00, 393.75) | 0.839 |
| ALB (g/L) | 27.75 (23.78, 33.20) | 31.00 (27.65, 35.48) | 33.30 (27.00, 37.50) | 37.25 (32.05, 40.55) | < 0.001 |
| TC (mmol/L) | 5.10 (4.01, 6.16) | 4.38 (3.89, 5.08) | 4.59 (3.77, 5.70) | 4.51 (3.82, 5.49) | 0.030 |
| TG (mmol/L) | 1.64 (1.23, 2.29) | 1.48 (1.09, 2.12) | 1.66 (1.27, 2.30) | 1.84 (1.34, 2.65) | 0.020 |
| LDL-C (mmol/L) | 3.43 (2.44, 4.23) | 2.77 (2.35, 3.52) | 2.96 (2.46, 3.68) | 2.84 (2.24, 3.76) | 0.017 |
| HDL-C (mmol/L) | 1.08 (0.87, 1.36) | 1.04 (0.88, 1.34) | 0.97 (0.79, 1.22) | 0.99 (0.84, 1.12) | 0.023 |
| NLR | 2.35 (1.71, 3.50) | 2.33 (1.94, 3.34) | 2.27 (1.70, 2.83) | 2.29 (1.62, 3.02) | 0.291 |
| 24-h UP, g/24 h | 5.22 (2.94, 9.94) | 3.87 (1.52, 6.81) | 3.45 (0.94, 7.33) | 1.57 (0.43, 3.42) | < 0.001 |
| TBil (μmol/L) | 3.40 (2.60, 3.80) | 5.55 (4.80, 6.20) | 8.10 (7.39, 9.30) | 12.70 (11.10, 15.48) | < 0.001 |

Data are presented as mean ± standard deviation (SD), median (interquartile range, IQR), or number (percentage), as appropriate..

**Abbreviations:** DN, diabetic nephropathy; BMI, body mass index; SBP, systolic blood pressure; DR, diabetic retinopathy; ACEI/ARB, angiotensin-converting enzyme inhibitors/angiotensin II receptor blockers; SGLT2i, sodium-glucose cotransporter-2 inhibitors; eGFR, estimated glomerular filtration rate; UA, uric acid; ALB, albumin; TC, total cholesterol; TG, triglycerides; HDL-C, high-density lipoprotein cholesterol; LDL-C, low-density lipoprotein cholesterol; NLR, neutrophil-to-lymphocyte ratio; 24-h UP, 24-hour urinary protein; TBil, total bilirubin.

**Supplementary Table S2**. Renal pathological characteristics according to TBil quartiles (μmol/L)

| **Variable** | **Q1**  **(≤ 4.20, n = 98)** | **Q2**  **(4.30–6.76, n = 102)** | **Q3**  **(6.80–10.37, n = 99)** | **Q4**  **(≥ 10.40, n = 102)** | ***p*** |
| --- | --- | --- | --- | --- | --- |
| Glomerular lesion classification |  |  |  |  |  |
| Class III/IV DN, n (%) | 76 (77.6) | 78 (76.5) | 64 (64.6) | 40 (39.2) | <0.001 |
| Global glomerulosclerosis (%), median (IQR) | 19.0 (7.0, 45.3) | 17.5 (7.0, 36.6) | 17.2 (6.7, 35.8) | 14.9 (0, 36.5) | 0.576 |
| IFTA scores, n (%) |  |  |  |  | 0.019 |
| 0/1 | 28 (28.6) | 43 (42.2) | 35 (35.4) | 46 (45.1) |  |
| 2 | 48 (49.0) | 42 (41.2) | 53 (53.5) | 47 (46.1) |  |
| 3 | 22 (22.4) | 17 (16.7) | 11 (11.1) | 9 (8.8) |  |
| Interstitial inflammation scores, n (%) |  |  |  |  | 0.004 |
| 0 | 15 (15.3) | 9 (8.8) | 12 (12.1) | 20 (19.6) |  |
| 1 | 61 (62.2) | 64 (62.7) | 66 (66.7) | 75 (73.5) |  |
| 2 | 22 (22.4) | 29 (28.4) | 21 (21.2) | 7 (6.9) |  |
| Arteriolar hyalinosis scores, n (%) |  |  |  |  | 0.251 |
| 0 | 24 (24.5) | 18 (17.6) | 12 (12.1) | 15 (14.7) |  |
| 1 | 41 (41.8) | 39 (38.2) | 43 (43.4) | 48 (47.1) |  |
| 2 | 33 (33.7) | 45 (44.1) | 44 (44.4) | 39 (38.2) |  |
| Arteriosclerosis scores, n (%) |  |  |  |  | 0.904 |
| 0 | 16 (16.3) | 17 (16.7) | 12 (12.1) | 13 (12.7) |  |
| 1 | 63 (64.3) | 62 (60.8) | 66 (66.7) | 70 (68.6) |  |
| 2 | 19 (19.4) | 23 (22.5) | 21 (21.2) | 19 (18.6) |  |

Data are presented as n (%) or median (interquartile range), as appropriate.

DN was classified according to the Renal Pathology Society (RPS) classification.

Tubulointerstitial and vascular lesions were semi-quantitatively scored according to the Renal Pathology Society classification.

**Abbreviations:** IFTA, interstitial fibrosis and tubular atrophy; TBil, total bilirubin.

**Supplementary Table S3.** Stratified analyses of the association between TBil (μmol/L) and advanced DN

| **Subgroup** | **OR (95% CI)** | ***P* for interaction** |
| --- | --- | --- |
| 24-h UP, g/24 h |  | 0.368 |
| ≤1 | 0.97 (0.83–1.10) |  |
| 1-3.5 | 0.89 (0.79–0.98) |  |
| ≥3.5 | 0.85 (0.76–0.95) |  |
| eGFR, mL/min/1.73 m^2^ |  | 0.128 |
| ≥60 | 0.85 (0.77–0.93) |  |
| <60 | 0.98 (0.88–1.09) |  |
| HbA1c, % |  | 0.731 |
| ≤7 | 0.87 (0.77–0.96) |  |
| ＞7 | 0.88 (0.81–0.95) |  |
| Duration of diabetes, years |  | 0.981 |
| ≤10 | 0.89 (0.81–0.96) |  |
| ＞10 | 0.85 (0.76–0.95) |  |

Data are presented as odds ratios (ORs) with 95% confidence intervals (CIs).

Estimates were derived from the fully adjusted multivariable logistic regression model (Model 3). *P* values for interaction were obtained by including multiplicative interaction terms in the model. Advanced DN was defined as Renal Pathology Society classes III–IV.

**Supplementary Table S4.** Sensitivity analysis of the association between TBil and advanced DN after excluding patients with documented diuretic use

| **Model** | **OR (95% CI)** | ***P*** |
| --- | --- | --- |
| Model 1 | 0.86 (0.81–0.91) | <0.001 |
| Model 2 | 0.85 (0.80–0.91) | <0.001 |
| Model 3 | 0.89 (0.83–0.95) | <0.001 |

Model 1 was adjusted for age, sex, BMI, smoking status, and duration of diabetes.

Model 2 further adjusted for SBP, HbA1c, as well as the use of ACEI/ARB and SGLT2i.

Model 3 defined as the fully adjusted model and additionally included serum UA, ALB, NLR, TC, TG, HDL-C, LDL-C, and ln-UP

Advanced DN was defined as Renal Pathology Society classes III–IV.

**Supplementary Table S5.** Sensitivity analysis of the association between TBil and DN pathological severity using ordinal logistic regression.

| **Variable** | **OR (95% CI)** | ***P*** |
| --- | --- | --- |
| TBil (μmol/L) | 0.90 (0.860–0.949) | < 0.001 |
| Age (years) | 1.01 (0.99–1.03) | 0.294 |
| Male, n (%) | 1.21 (0.75–1.97) | 0.438 |
| BMI (kg/m²) | 1.05 (0.95–1.16) | 0.303 |
| Duration of diabetes (years) | 1.05 (1.02–1.08) | 0.002 |
| SBP (mmHg) | 1.00 (0.99–1.01) | 0.842 |
| Cigarette smoking, n (%) | 1.09 (0.68–1.73) | 0.726 |
| HbA1c (%) | 1.07 (0.95–1.20) | 0.276 |
| ACEI/ARB | 0.90 (0.61–1.32) | 0.578 |
| SGLT2i, n (%) | 1.02 (0.68–1.53) | 0.916 |
| UA (µmol/L) | 1.00 (0.99–1.01) | 0.153 |
| ALB (g/L) | 0.97 (0.92–1.01) | 0.142 |
| TC (mmol/L) | 1.08 (0.88–1.33) | 0.462 |
| TG (mmol/L) | 1.00 (0.98–1.02) | 0.691 |
| HDL-C (mmol/L) | 0.61 (0.35–1.08) | 0.089 |
| LDL-C (mmol/L) | 0.88 (0.70–1.11) | 0.289 |
| NLR | 1.00 (0.90–1.13) | 0.953 |
| 24-h UP, g/24 h | 1.65 (1.32–2.07) | < 0.001 |

Ordinal logistic regression was performed with DN pathological classes I–IV as the outcome.

All estimates were derived from the fully adjusted multivariable model (Model 3).

Odds ratios represent cumulative odds ratios per one-unit increase in continuous variables. Because the proportional odds assumption was violated for serum total bilirubin, as assessed by the Brant test, these ordinal regression results should be interpreted cautiously and are provided for sensitivity analysis purposes only.
